# Performance of magnetic field‐guided navigation system for interventional neurosurgical and cardiac procedures

**DOI:** 10.1120/jacmp.v6i3.2111

**Published:** 2005-08-17

**Authors:** James C.H. Chu, Wen Chien Hsi, Lincoln Hubbard, Yunkai Zhang, Damian Bernard, Pamela Reeder, Demetrius Lopes

**Affiliations:** ^1^ Department of Radiation Oncology Rush University Medical Center Chicago Illinois 60612 U.S.A.; ^2^ Department of Medical Physics Rush University Medical Center Chicago Illinois 60612 U.S.A.; ^3^ Department of Clinical Engineering Rush University Medical Center Chicago Illinois 60612 U.S.A.; ^4^ Department of Neurosurgery Rush University Medical Center Chicago Illinois 60612 U.S.A.

**Keywords:** magnetic field navigation, interventional radiology, neurosurgery

## Abstract

A hospital‐based magnetic guidance system (MGS) was installed to assist a physician in navigating catheters and guide wires during interventional cardiac and neurosurgical procedures. The objective of this study is to examine the performance of this magnetic field‐guided navigation system. Our results show that the system's radiological imaging components produce images with quality similar to that produced by other modern fluoroscopic devices. The system's magnetic navigation components also deflect the wire and catheter tips toward the intended direction. The physician, however, will have to oversteer the wire or catheter when defining the steering angle during the procedure. The MGS could be clinically useful in device navigation deflection and vessel access.

PACS numbers: 07.55.Db, 07.85.‐m

## I. INTRODUCTION

Conventional interventional cardiac and neurosurgical procedures are performed manually by using guide wires and special catheters under fluoroscopic guidance. The ability and experience of a physician to navigate and track a guide wire or a catheter through tortuous vasculature are important to the success of these procedures. However, the deflection that can be achieved at the tip of a conventional guide wire or a catheter may be limited by the tortuosity of blood vessels and the orientation of the catheter. It requires a great deal of skill to torque the device and reach the site of the lesion. In addition, the radiation exposure to both patients and physicians during long procedures may be a limiting factor.^(^ ^1^ ^–^ ^3^ ^)^

Externally applied magnetic fields that control the movement and position of magnetically tipped instruments can potentially facilitate these procedures.^(^ ^1^ ^,^ ^4^ ^–^ ^8^ ^)^ A magnetic guidance system (MGS) (Stereotaxis, Inc., St. Louis, MO) consisting of three orthogonal superconducting magnetic coils and a biplanar fluoroscope has been approved for human clinical trials and was installed for such procedures in our institution. The physician specifies the desired orientation of the tip of the guide wire or catheter through two orthogonal fluoroscopic views produced by a pair of flat panel detectors. The navigation system then calculates and applies the desired currents to the three electromagnets. Specially designed guide wires and catheters with magnetic tips are used with the MGS for the purpose of magnetic deflection and vessel access. Once the desired tip orientation is confirmed by fluoroscopy, the physician then advances the wire or catheter manually. This process is repeated whenever the wire tip reaches another difficult‐to‐turn position in the vasculature. This study reports the results of performance tests of digital fluoroscopy and the navigation system in conjunction with the Stereotaxis magnetic guide wire and microcatheters.

### II. MATERIALS AND METHODS

**Figure 1:**
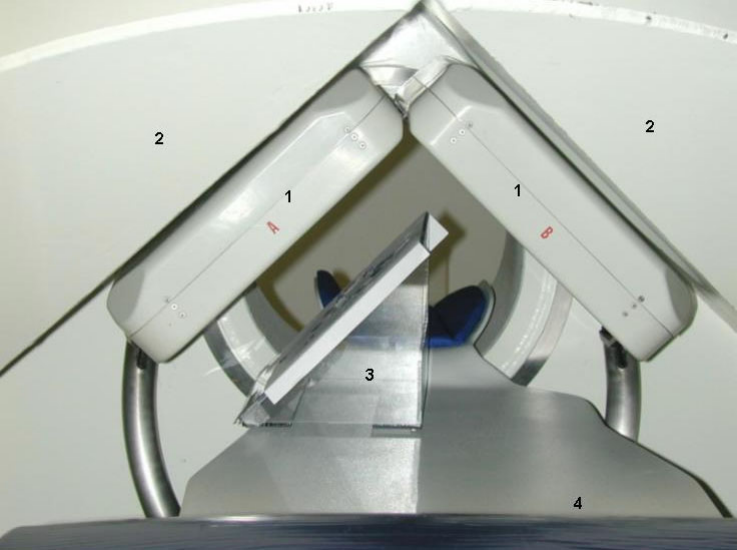
Experimental setup showing the surface of the phantom (3) on the table (4) is parallel to the flat panel imager (1) on the left. Superconducting coils (2) are located above each flat panel and around the opening at the center. The catheters and guide wire are placed on the phantom surface during the experiment.

The MGS has three orthogonal superconducting magnetic coils under the cover, a magnetically shielded X‐ray tube, and two orthogonal fluoroscopic flat panel detectors of $20\times 20\;{\text{cm}}^{2}$ (Fig. 1). The detectors contain a minimum number of electronic components with a ferrous core and are designed to be magnetically compatible. The manufacturer of both the magnet system and fluoroscope system is Stereotaxis, Inc. However, Varian Medical Systems (Palo Alto, CA) provides major components of the fluoroscopic imaging chain (Varian PaxScan 2520).

The American Association of Physicists in Medicine (AAPM) fluoroscopic imaging test procedures, with minor modification, were used to determine the quality of the imaging system used to visualize the magnetic tip.^(^ ^9^ ^,^ ^10^ ^)^ The purpose was to determine whether the fluoroscopy system is compliant with the conventional standard, or whether it had extraordinary capabilities or required unusual operation.

The low‐contrast resolution was tested by placing a thin (0.8 mm) aluminum (1800 alloy) sheet with a series of five holes ranging from 1.6 mm to 6.4 mm in diameter between two thicker (each 19 mm thick) uniform pieces of 1800 alloy aluminum (Nuclear Associates Penetrometer, Model 07‐706). These combined sheets were placed at right angles to, and completely covering, the fluoroscopy beam, at about 30 cm in front of the image intensifier (II) surface. The fluoroscopy unit was operated at 15 fps in its standard auto brightness mode, and the images were evaluated from the standard monitor. The evaluation was carried out at both the regular and the “zoom” viewing. The contrast resolution was scored based on the number of holes clearly visible. The high‐contrast spatial resolution was tested by using a series of brass mesh patterns (RMI Model 141) with the two 19‐mm thick aluminum plates of the penetrometer.^(^ ^11^ ^)^ Positioning, operation of the fluoroscopy, and image evaluation were essentially the same as used in the contrast resolution testing. The dose rate was determined by a calibrated ion chamber system (Victoreen model 4000M+). The ion chamber was placed 30 cm in front of the II face.

**Figure 2:**
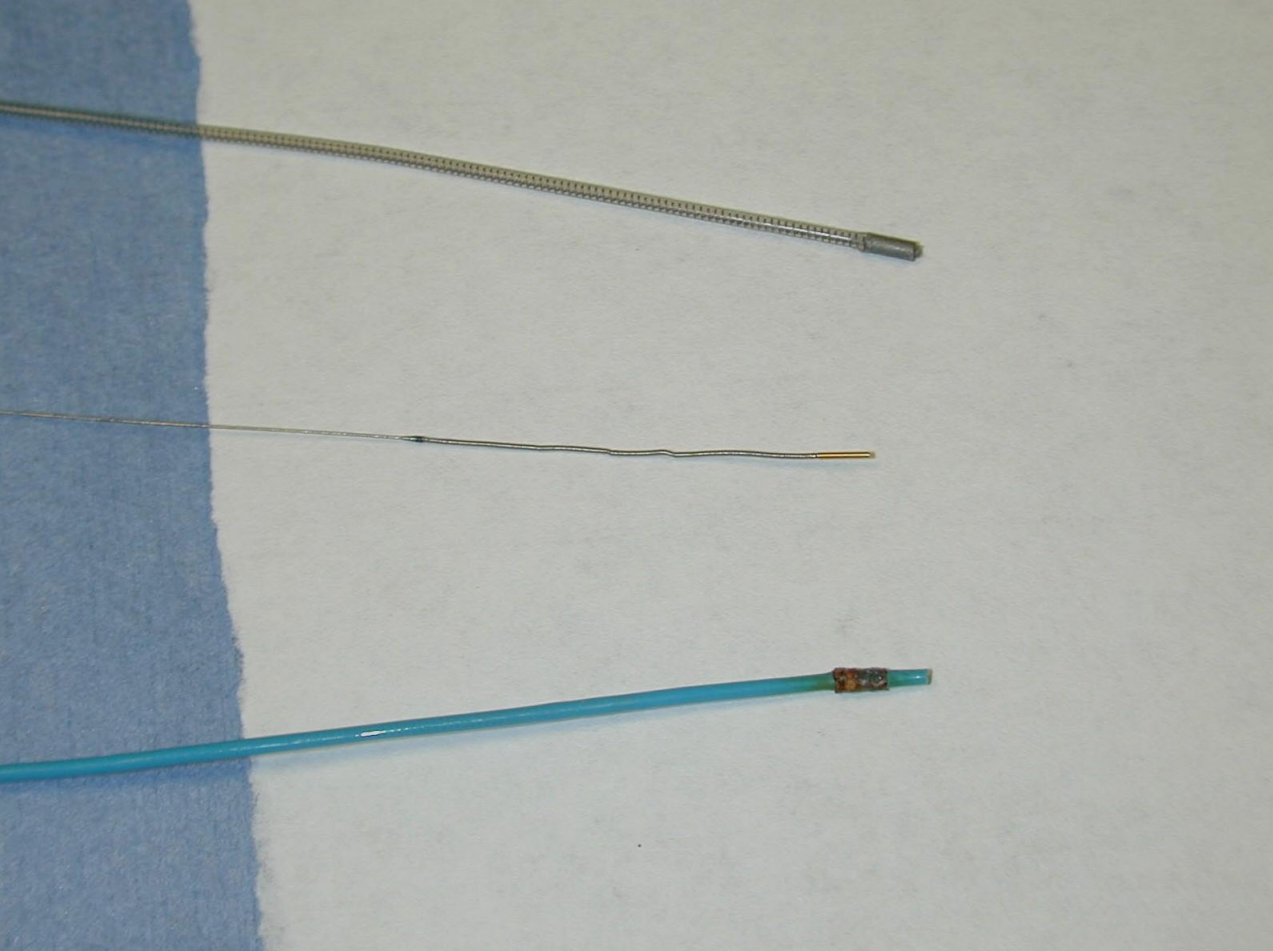
Guide wire and catheters used in the experiment. The outside diameter for the microcatheters (top and bottom) is 0.035“ (2.6 Fr). The guide wire (middle) has a diameter of 0.014”. The magnetic tips of all three devices are clearly visible.

The steering capability of the MGS was tested with a magnetic guide wire and two magnetic catheters (Fig. 2). These special‐purpose wire and catheters have small neodymium iron boron magnets imbedded within their distal ends. The magnetic steering is performed in a navigation volume of $14\times 11\times 13\;{\text{cm}}^{3}$ centered at the isocenter of the system. The positions of the wire or catheters within the navigation volume are visible from a pair of orthogonal X‐ray images. These images are produced by a biplane X‐ray system with two flat panel detectors. A vector drawn on these images defines the desired orientation of the tips. The vector color indicates whether the device tip is within the navigation volume. Since the magnetic field can be activated only when the device is within the navigation volume, the user should adjust the patient couch position and assure that the magnetic tip is close to the isocenter during the procedure. Once the desired vector orientation is accepted, the MGS then activates three sets of orthogonal superconducting magnetic coils to achieve the required magnetic field to steer the magnetic tip. A sheet of polar coordinate graph paper taped on a flat surface phantom was used for this experiment. Radio‐opaque markers were placed at the center and at the 0°, 15°, 30°, 60°, 90°, and 135° positions on the graph paper. The guide wire and the catheters were placed on the phantom, one at a time, and were allowed to pivot at 2 cm from the tip. The pivot point was fixed at the center of graph paper. The requested steering angles were compared with that achieved at two planes parallel to the two flat panels. The panels were positioned at 45° from the patient table. The distance between the phantom surface and the flat panel is about 10 cm. The experimental setup for one such measurement is shown in Fig. 1. Additional measurements were made with the phantom surface at 45° to the flat panels (parallel to the table top) and close to the center of the navigation volume.

**Figure 3:**
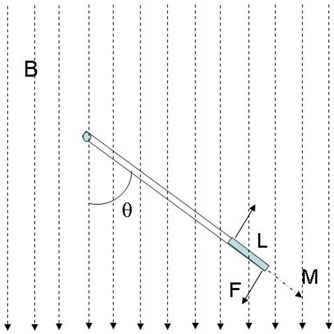
Magnetic field‐induced device tip deflection.

The defection torque can be written as^(^ ^1^ ^,^ ^2^ ^)^

(1) $$\vec{\tau }=\vec{M}\times \vec{B}•AL,$$

where $\vec{\tau }$ is the deflection torque applied at the tip of the guide wire or catheter, $\vec{M}$ is the magnetization vector of that magnetic tip, $\vec{B}$ is the applied external magnetic field, *A* is the cross‐sectional area of the tip, and *L* is the length of the tip. From the above equation, we have the magnitude of the deflection torque τ=MALBsin(θ), where θ is the angle between the magnetic field and the magnetization vector, as shown in Fig. 3. Torque can also be described by two identical forces *F* acting in opposite directions on the two ends of the magnet:

(2) $$\tau =2\frac{L}{2}F.$$

Thus, we have the force acting on the distal end of the magnet tip:

(3) F=MABsin⁡(θ).

A DC Manetometer (Alpha Lab, Salt Lake City, UT) was used to measure magnetic field components both along and perpendicular to the catheters or guide wire during the experiment. All magnetic field measurements are performed on the surface of the phantom.

## II. RESULTS AND DISCUSSION

At 15 fps, the “average patient skin dose air kerma rate” (i.e., the air kerma for the technique factors elicited by 38.8 mm of 1800 alloy aluminum) was 22.3 mGy/min (2.5 R/min). This is within the 1 to 4R/R range expected for the continuous mode of fluoroscopy study.^(^ ^9^ ^)^ This is also about half of the “suggested” 5R/R maximum, one‐fourth of the mandatory 10R/R general purpose unit limit, and one‐eighth of the “high dose rate” limit.^(^ ^12^ ^)^ The spatial resolution varies from about 16lp/lp to 22lp/lp, depending on the magnification setting. The manufacturer's specification, however, is 20lp/lp for all fields‐of‐view (FOVs), since its digital zoom simply replicates pixels and does not improve resolution. Nevertheless, the measured results are similar to the 18lp/lp to 20lp/lp achievable from a modern fluoroscopy unit.^(^ ^9^ ^)^ The contrast resolution is just adequate to visualize the $1.6\;\text{mm}(1/16^{\prime \prime })$ hole in the penetrometer plate. This low‐contrast result is typical of modern fluoroscopy units.^(^ ^9^ ^)^ Overall, the performance of the fluoroscopy system can be described as close to the performance of a modern conventional unit running in normal operation.

**Figure 4:**
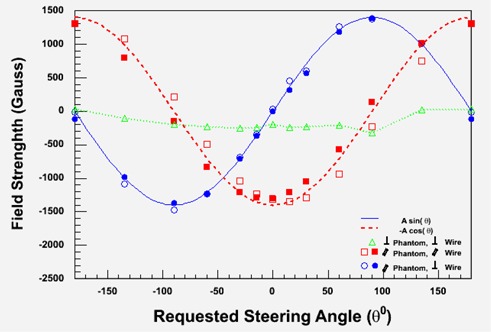
Magnetic field measured with phantom surface parallel to the flat panel imagers. The open and solid symbols are measured with the phantom surface parallel to the left and right flat panel detectors, respectively. The square symbols are for magnetic field parallel to the catheters or wire, and the circles are data points measured in the perpendicular direction. The as expected, that a sine function fits all data measured along the catheter (square symbols). Another sine function with a 90° phase offset describes the data measured in the perpendicular direction (circle symbols). The magnetic field component perpendicular to the phantom surface does not change with steering angle (triangle symbols).

Figure 4 shows the variation of magnetic field under various steering conditions. The open and solid symbols are measured with the phantom surface parallel to the left and right flat panel detectors, respectively. The square symbols are for magnetic field parallel to the catheters or wire, and the circles are data points measured in the perpendicular direction. The figure shows, as expected, that a sine function fits all data measured along the catheter (square symbols). Another sine function with a 90° phase offset describes the data measured in the perpendicular direction (circle symbols). The magnetic field component perpendicular to the phantom surface does not change with steering angle (triangle symbols). A relatively constant magnetic field perpendicular to the phantom surface confirms that the steering is only performed on a plane parallel to the phantom surface.

Table 1 shows the achieved steering angles for the guide wire and catheters under various experimental conditions. The prescribed steering angles range from 15° to 135° in both positive (toward patient's head) and negative (toward patient's feet) directions. The data in parentheses are the achieved angles in the negative direction. Generally, greater steering angles are achieved in the positive direction. Neither the catheters nor the guide wire achieved the requested steering angles. The amount of magnetic field‐induced deflection of the stiffer catheter is less than that of the other magnetic catheter or guide wire, as would be expected based on the catheter shaft design differences. The ultimate steering angle achieved is dependent on the strength and uniformity of the neodymium magnet at the tip, the externally applied magnetic field, the stiffness of the wire or catheter, and the resistance of the medium. The magnetic field‐induced deflection in patients is anticipated to be less than the results recorded here because our experiment was conducted in air. This underdeflection is not likely to cause a problem clinically because the tip orientation is always checked under fluoroscopy before the device is advanced manually. The degree of steering in the negative direction is less than that in the positive direction in almost all cases. This may be due to magnetic field nonuniformity produced by the neodymium magnet at the tip and possible field asymmetry produced by the navigation system.

**Table 1:** Comparison of requested and achieved steering angle. The data in parentheses are the achieved angles in the negative direction.

Prescribed angle	15°	30°	60°	90°	135°
Achieved angle					
parallel to flat panel					
stiff catheter	5 (—)	12 (—)	29 (—)	50 (—)	—(—)
guide wire	10 (8)	21 (13)	44 (36)	72 (63)	108 (107)
flexible catheter	10 (9)	23 (19)	50 (39)	77 (60)	111 (112)
45° to flat panel					
flexible catheter	—	24 (23)	49 (45)	75 (71)	115 (113)

Recent studies have demonstrated the value of the MGS in interventional procedures. For example, Faddis et al. successfully navigated magnetic catheters to 51 predefined targets in 6 animal hearts.^(^ ^1^ ^)^ Ernst et al. performed MGS‐guided ablation of atrioventricular nodal reentrant tachycardia in 42 patients.^(^ ^14^ ^)^ Shiemann et al. compared MGS and conventional navigation techniques at 129 targeted turns in two phantoms; they found that the MGS technique has a higher success rate and reduced procedure and fluoroscopy times.^(^ ^2^ ^)^ A total of 30 patients were entered in a clinical trial using the MGS in our institution. The data presented here and our preliminary clinical impression confirm those earlier reports^(^ ^1^ ^–^ ^2^ ^,^ ^13^ ^,^ ^14^ ^)^ that magnetic steering technology may have the potential of improving vascular catheterization, reducing procedure time, and lowering radiation exposure.

## IV. CONCLUSION

Although the MGS could not deliver the requested steering angles, the system does in general deflect the wire or catheter tips in the intended direction. The MGS, therefore, could be clinically useful in device navigation deflection and vessel access for those patients treated with this unit. The physician would have to oversteer the wire or catheter when defining the steering angle on the flat panel images during the procedure. In addition, it is necessary that tip deflection be verified fluoroscopically before the device is manually advanced through the vasculature. The MGS appears to be safe and useful when used under these conditions with properly trained personnel. Additional measurements, however, should be made periodically to assure the stability of the MGS and the reproducibility of the achieved steering angles.

## ACKNOWLEDGMENT

The authors wish to thank John Kinder of Stereotaxis, Inc., for providing information on the magnetic field‐guided navigation system.
